# Family Resource Dilution in Expanded Families and the Empowerment of Married Only Daughters: Evidence From the Educational Investment in Children in Urban China

**DOI:** 10.3389/fpsyg.2021.610482

**Published:** 2021-02-09

**Authors:** Xiaotao Wang, Xiaotian Feng

**Affiliations:** ^1^School of Social Development, Nanjing Normal University, Nanjing, China; ^2^School of Social and Behavioral Sciences, Nanjing University, Nanjing, China

**Keywords:** family resource dilution, only daughters, educational investment, China, Chinese expanded families

## Abstract

The One-Child Policy dramatically changed the Chinese family structure, and the literature indicates that only children may have an advantage in terms of family resource dilution. Moreover, as Chinese families traditionally prioritize investing in sons, only daughters are found to have been empowered by the policy because they did not need to compete with their brothers for parental investment. However, the literature is limited to only teenage children when they were still living in their parents' homes. It is unclear whether—when the generation of only children grew up and married—their family structure differed from that of children with siblings and whether married only daughters retained more family resources from their parents. Based on the data analysis of a 2016 survey, “Study of Youths in 12 Cities of Mainland China,” including a sample of 1,007 fathers and 2,168 mothers born between 1975 and 1985, this study explores the empowerment of married only daughters, employing the theory of family resource dilution in expanded Chinese families. Using educational investment in children as an example, and with random intercept models, this study presents empirical evidence that the dilution of family resources in Chinese expanded families still benefits males and patrilineal practices. Thus, this study demonstrates that Chinese families still tend to sacrifice the interests of married daughters to ensure support for their adult sons. However, it also illustrates that married only daughters could still connect to their parents' resources, giving them a relatively dominant position for decision-making regarding the family's educational expenditure on her own children. Thus, this study extends our understanding of the family resource dilution theory to Chinese expanded families, underscoring the need for further research on Chinese only children after they marry and form families of their own.

## Introduction

The Chinese government promoted the One-Child Policy (OCP) in the late 1970s and early 1980s to accelerate China's socioeconomic modernization through birth control (Greenhalgh, 2008). This policy remained in force, primarily in urban areas (Guo et al., 2003) until 2016, when it was modified to a Universal Two-Child Policy (Wang et al., 2016). China has made remarkable socioeconomic achievements over the past 30 years, becoming the world's most extensive one-child population due to the OCP (Peng, 2011; Wang et al., 2012). It is estimated that there were more than 220 million only children in mainland China at the end of 2015 (Li et al., 2018). Moreover, although the policy was modified in 2016 to allow two children per couple, most young urban couples still choose to have only one child (Feng, 2018). Indeed, while facilitating China's economic development, the OCP appears to have significantly reduced the country's fertility (Feng et al., 2014). As such, only children were, and continue to be, one of the most important demographic groups in China's population (Feng, 2020).

The OCP and the great number of only children have brought about important changes in Chinese family structure (Feng et al., 2014). The size of Chinese families declined quickly (Peng and Huang, 1996), and a three-member family pattern rapidly became the dominant family type in urban China (Feng, 1992). The intra-family relationships in only-child families tend to be simple as the parents choose to put all their energy and resources into providing for their one child (Fong, 2004). Researchers have expressed concern regarding the development of only children in such families, considering whether Chinese only children would be “spoiled” and become “Little Emperors” (Falbo, 1996; Feng, 2000; Cameron et al., 2013). However, limited evidence has been produced that indicates such a difference between only and non-only children (Poston and Falbo, 1990; Falbo and Poston, 1993; Falbo, 1996; Feng, 2000; Edwards et al., 2005; Zhao et al., 2013; Falbo and Hooper, 2015; Song and Wang, 2019). Rather, these studies focused on the personality development of only children while failing to examine the lives of children within their family units (Fong, 2004; Short, 2005).

Family has a significant influence on only children (Jack, 2000; Wei et al., 2016; Xu, 2017). One of the significant impacts of the OCP on Chinese families is that only children enjoy all the resources of their parents and family, which relates to the theory of the family resource dilution paradigm (Blake, 1981). Based on the rational action theory, the family resource dilution paradigm assumes that the family resources available to children—such as economic resources and parental care—are always limited. According to this paradigm, the more children there are in a family, the fewer resources are available for each child, resulting in lower individual attainments (Blake, 1981). Only children have the most obvious advantage, as there are no competitors in the family, whereas for children with siblings, the number of siblings makes a notable difference (Blake, 1981). Although unobserved factors in the family may result in a false correlation (Downey et al., 1999; Guo and VanWey, 1999), many studies have found empirical evidence of a significant negative correlation between the number of siblings and child attainments (Blake, 1981; Downey, 1995; Steelman et al., 2002; Sandberg and Rafail, 2014; Öberg, 2015; Gibbs et al., 2016). The competitive paradigm confluence theory suggests that only children may develop certain weaknesses due to not having grown up with teaching other children in the family; however, this theory also indicates that the family environment has a clear dilutive effect based on the number of children in a family unit (Zajonc and Markus, 1975; Chen, 2015).

The family resource dilution effect holds in the Chinese context, where it is further influenced by gender. Chu et al. (2007) demonstrated that such dilution had a greater impact on daughters than sons in Taiwan, Province of China. Traditional Chinese families have been characterized as vertical and patrilineal (Thornton and Lin, 1994; Fei, 1998), with the male line valued over the female line since daughters will join another family via marriage (Hsu, 1972). Consequently, parents are more likely to want sons (Li et al., 2011) and tend to support their sons over their daughters—males generally receive greater investment than their female siblings (Short et al., 2001; Hu and Tian, 2018). Concurrently, scholars of family resource dilution in China have also noted the possible impact of the OCP (Fong, 2004). It has been argued that the gendered family resources dilution has been challenged by the OCP, which restricts families in urban China to only one child regardless of gender. Accordingly, parents in only-daughter families have “no choice” but to invest in their daughter and tend to have high expectations of them, as a result (Fong, 2002, 2004). Indeed, nationwide survey data demonstrate gender inequalities in educational attainment and the return to education among siblings, but no such gender inequality among only children (Wang, 2011a,b). A study of Chinese college students revealed that only daughters exhibit the highest visual imagination abilities and creative problem solving (Guo et al., 2018). Thus, urban only daughters appear to have been “empowered” by the OCP (Fong, 2004).

The preceding literature highlights the impact of the OCP from the perspective of family, indicating that only children have an obvious advantage in terms of family resource sharing and that only daughters may have bypassed the traditional preference for sons in Chinese families and are “empowered” by the policy (Fong, 2004). However, the literature has primarily focused on teenage only children when they were living with their parents (Feng, 2002). However, the first generation of Chinese only children—that is, only children born in the late 1970s and early 1980s, at the beginning of the implementation of the OCP (Feng, 2005)—have grown up and formed their own families (Liu, 2008). This raises questions regarding whether married only children will continue to have an advantage in the dilution of family resources and whether married only daughters will retain their position of empowerment as adults with families of their own.

In contrast to western nuclear family units (Parsons, 1943), Chinese families are treated as “expanded families” (Fei, 1998). The majority of grown-up children, especially males, will keep in close contact with their parents (Lee et al., 1994; Xu et al., 2019), with many continuing to live in their parents' homes in adulthood (Xie and Zhu, 2009; Yasuda et al., 2011). While the parents are relatively young and do not need much care, the main intergenerational exchange content is the parents' continued support for their children (Liu, 2012). Typically, Chinese parents help their married children care for their own children (Chen et al., 2011). For instance, in Xiamen, a city in the southeast of China, 45.4% of grandparents help with the grandchildren's education, working closely as joint caregivers with the parents (Goh, 2011).

In this process, family resources, including both parents' resources and grandparents' resources, are important. When parents have higher education and better income, or in other words, better cultural capital and economic capital, their children's education will benefit from it (Bourdieu and Passeron, 1990; Li, 2006; Jæger, 2011). One of the biggest goals of Chinese families in saving money is to educate their children (Wei and Zhang, 2011). Simultaneously, grandparents with better resources will also positively impact the grandchildren's education through the parents (Jæger, 2012; Hancock et al., 2016). Research from China has demonstrated that grandparents with better education and income will lead to higher educational attainment for their grandchildren (Zeng and Xie, 2014; Chiang and Park, 2015).

Therefore, in this study, the family's educational investment in children will be considered when discussing the family resource dilution in Chinese expanded families and the empowerment of married only daughters. As education is considered a core value of Chinese families, parents are highly concerned with their children's schooling (Lan, 2018). It has been suggested that Chinese families concentrate all their resources on their children's education, and as mentioned above, many grandparents are highly involved in the process (Goh, 2011). Particularly in Chinese cities, grandparents' support is of growing significance due to the increasing expenditure of certain kinds of education (Lin, 2018). Thus, in Chinese expanded families, the grandparents' resources are important to the educational investments in the children, and there is reason to believe that the theory of the dilution of resources is applicable in this situation. That is, the more siblings the parents have, the less support they will receive from their parents (the grandparents). Based on this theory, the first hypothesis is proposed—H1: the more siblings the parent has, the less their children will receive in education expenditure.

The preceding literature argues that Chinese families, traditionally, exhibit a preference for sons in their resources distribution. This is especially detrimental for daughters who are considered to lose their “family membership” when they marry (Hsu, 1972). Therefore, a high number of brothers would hypothetically have a greater negative effect than a high number of sisters—particularly for a married daughter—as male siblings would increase the competition for the family resources. Thus, the second hypothesis is proposed—H2: the influence of the number of brothers is greater than that of the number of sisters; when the mother has more brothers, she will have less investment in her children as a result.

Along with gender, seniority will also have a significant impact, as Chinese families traditionally emphasize the older sibling's duty to care for their younger siblings. For instance, in Taiwan, empirical research has identified that Chinese families often sacrifice the resources of older sisters to ensure the resources of younger brothers (Chu et al., 2007). Therefore, the third hypothesis of this study is proposed—H3: when the mother has younger brothers, her parents' resources are more likely to be spent on her younger brothers, which, in turn, would reduce the educational investment in her own children.

Only children are the exception to the scenarios explained above. As there is no competitor in the immediate family unit, only children can easily connect with their parents' resources—regardless of gender and both before and after marriage. Therefore, the fourth hypothesis proposes—H4: married only daughters will have an advantage over those with siblings in securing their family resources.

## Method

### Sample

This study analyzes data from “Study of Youths in 12 Cities of Mainland China,” a large survey conducted in early 2016 by the authors in collaboration with other researchers. The survey's research protocol was reviewed and approved by Nanjing University's Sociology Department ahead of data collection. Furthermore, administrators of the kindergartens and primary and junior middle schools where the data were collected approved the participants' protection through a Human Subjects Protocol.

The survey respondents were randomly selected using a stratified three-stage probability sampling procedure (Feng, 2017). In the first stage, 12 Chinese cities, including municipalities, capitals, and major, and medium/small cities in all eastern, central, and western regions, were selected using stratified sampling. In the second stage, different types of schools (i.e., kindergarten, primary, and junior middle schools) were randomly selected in each of the chosen cities, using simple random sampling. In the third stage, 3–6 classes were randomly chosen within each of the selected schools; cluster sampling was then used to select students, the parents of whom constituted the survey respondents.

All respondents were invited to fill out the self-administrated questionnaire, which took ~30 min to complete. With the teachers' help, the selected students were asked to take the questionnaires home with them to their parents. In this respect, students were asked to briefly introduce the survey's purpose and provide instructions for the questionnaire's completion. Accordingly, the respondents completed the questionnaires at home, and students returned the completed questionnaires to their teachers the following day.

Over 8,000 questionnaires were distributed, and 7,710 completed questionnaires were returned. Respondents comprised 2,567 fathers and 5,143 mothers. In line with prior research (Feng, 2000), this study limited respondents to the first generation of Chinese only and non-only children within the same cohort, namely, those born between 1975 and 1985, during which time the OCP was implemented. Thus, 1,348 fathers and 2,599 mothers were excluded due to not fulfilling these requirements. Responses containing incomplete or missing data (588 cases) were also excluded. Accordingly, the final sample for this study comprised 1,007 fathers and 2,168 mothers (via 3,175 students).

### Measures

To examine educational investment in children, the respondents were asked to report how much CNY had been spent on the education of the child who had brought home the questionnaire during the previous year. Educational investment was measured in two respects: (1) the expenses accrued by their child at school; and (2) expenses on their child's education outside of school, mainly for tutoring classes, which are popular in urban China. This study then constructed a dependent variable based on the total spending—that is, by combining the two types of educational expenditure, regardless of where it had been spent.

This study uses parents' sibship size as the key explanatory variable. Parents' sibship size was measured by their number of brothers or sisters. This measure can be further divided into two variables, namely, father's sibship size and mother's sibship size. For analytical purposes, these two variables were further distinguished by gender, resulting in the construction of four variables: father's number of brothers, father's number of sisters, mother's number of brothers, and mother's number of sisters. This study expanded these variables to include the following: father's number of elder brothers, father's number of younger brothers, father's number of elder sisters, father's number of younger sisters, mother's number of elder brothers, mother's number of younger brothers, mother's number of elder sisters, and mother's number of younger sisters. This study also examined the only-child effect, with the following two dummy variables included in the analysis: whether the father/mother was an only child or not; and an only child was coded 1, while a child with siblings was coded 0.

This study included the following group of controlling variables: parents' resources, grandparents' resources, and children's information. Parents' resources comprised six variables: parents' age, registered residence, education, occupation, income, and housing. More specifically, age was measured by the parents' average age. Registered residence was measured using a dummy variable: both parents being from a rural residence was coded 1, while at least one parent being from an urban residence was coded 0. Education was measured by the total number of years of completed education of the more educated parent, regardless of schooling type. Occupation was measured using a dummy variable: both parents employed in a non-monopoly industry was coded 0, while at least one parent employed in a monopoly industry was coded 1. Income was measured by the higher monthly income of the father or mother. Finally, housing was dichotomous, indicating whether the parents owned their own houses or not.

Grandparents' resources comprised six variables: education of the father's parents, education of the mother's parents, help from the father's parents, help from the mother's parents, coresidence of the father's parents, and coresidence of the mother's parents. The education of the father/mother's parents was measured by the more educated grandparent's total number of years of completed education. Help provided by the father/mother's parents was measured by whether they assisted with housework and child care. The coresidence variables were dummy variables: grandparents co-residing with parents was coded 1, while grandparents not co-residing with parents was coded 0.

Children's information comprised five variables: sibling, age, gender, school type, and expected years of schooling. The first variable, sibling, was categorical, indicating how many siblings a child had if any. Child's gender was a dummy variable, with males constituting the reference group. School type consisted of three categories: kindergarten, primary school, and junior middle school, and was measured by dummy variables, with kindergarten used as the reference group. Finally, expected years of schooling was measured by the number of years that the parent expected their child to complete.

### Statistical Analysis

Families in the same city may be subject to unobservable city-level characteristics as each city's educational environment may differ. Accordingly, this study employed random intercept models to mitigate this issue. First, descriptive statistics were derived to illustrate the means or percentage of all variables, as well as the average educational investment in children according to parents' sibship size. Second, random intercept models were used to estimate the effects of parents' sibship size on the educational investment in their children and to further distinguish the effects of sibship size by gender. Third, this study added another variable—namely, parents of only children or parents of children with siblings—to the model to explore the only-child effect of parents on educational investment in children. All data analyses were conducted using STATA 15.

## Results

### Descriptive Analyses

Table 1 presents the means or percentages of the study variables, making simple comparisons regarding whether the fathers/mothers are only children or children with siblings. This study's dependent variable—educational investment in children—is presented in the first row. The results indicate that the educational cost of families in urban China, for our sample, is fairly high—one family spent an average of CNY 19,210 (1 CNY is worth about 1/7 US dollar) per year on the education of one child. In comparison, the total annual expenditure per person in urban China in 2015 was CNY 21,392 (National Bureau of Statistics of China, 2019). As expected, the results demonstrate that the families in the sample seemed to spend more on children's education when the parents were only children (fathers, CNY 23,230; mothers, CNY 25,080) compared to those who had siblings (fathers, CNY 17,710; mothers, CNY 17,350), and the *t*-tests demonstrated that these differences were significant [*t*_(3173)_ = 9.40, *p* = 0.000 for fathers, and *t*_(3173)_ = 12.85, *p* = 0.000 for mothers].

**Table 1:** Descriptive statistics.

	**Total (3,175)**	**Father**		**Mother**	
		**Only child (861)**	**Child with siblings (2,314)**	**Only child (763)**	**Child with siblings (2,412)**
Educational investment (Thousand Yuan, CNY)	19.21	23.23	17.71	25.08	17.35
Fathers' siblings	1.31	0	1.79	0.79	1.47
Mothers' siblings	1.42	0.80	1.66	0	1.87
Fathers' brothers	0.57	0	0.78	0.34	0.64
Fathers' sisters	0.74	0	1.02	0.45	0.83
Mothers' brothers	0.73	0.43	0.85	0	0.97
Mothers' sisters	0.69	0.37	0.81	0	0.91
**Parents' resources**					
Age (year)	36.78	35.83	37.13	36.22	36.95
Registered residence					
Rural (%)	42.30	19.51	50.78	16.51	50.46
Urban (%)	57.70	80.49	49.22	83.49	49.54
Schooling (year)	14.39	15.47	13.99	15.85	13.93
Occupation					
Monopoly industry (%)	51.46	64.11	46.76	65.66	46.97
Non-Monopoly industry (%)	48.54	35.89	53.24	34.34	53.03
Income (Thousand Yuan, CNY)	6.97	7.65	6.71	8.38	6.52
Housing					
Own House (%)	68.03	68.87	67.72	73.29	66.21
Others (%)	31.97	31.13	32.28	26.21	33.79
**Grandparents' resources**					
Fathers' parents' schooling (year)	9.81	11.29	9.27	10.99	9.44
Mothers' parents' schooling (year)	10.06	10.99	9.71	11.51	9.60
Fathers' parents' help					
Yes (%)	33.86	44.02	30.08	32.37	34.33
No (%)	66.14	55.98	69.92	67.63	65.67
Mothers' parents' help					
Yes (%)	21.64	22.76	21.22	42.33	15.09
No (%)	78.36	77.24	78.78	57.67	84.91
Fathers' parents' coresidence					
Co-residing (%)	34.58	38.91	32.97	24.25	37.85
No Co-residing (%)	65.42	61.09	67.03	75.75	62.15
Mothers' parents' coresidence					
Co-residing (%)	13.57	14.29	13.31	30.01	8.37
No Co-residing (%)	86.43	85.71	86.69	69.99	91.63
**Children's information**					
Gender					
Male (%)	49.13	48.32	49.44	46.00	50.12
Female (%)	50.87	51.68	50.56	56.00	49.88
Age (year)	8.46	7.20	8.93	7.17	8.87
Sibling					
0 (%)	72.94	81.53	69.75	80.73	70.48
1 (%)	25.17	18.23	27.74	19.00	27.11
2 (%)	1.89	0.23	2.51	0.26	2.40
School					
Kindergarten (%)	33.54	48.20	28.09	50.46	28.19
Primary school (%)	55.53	48.20	58.25	45.61	58.67
Junior middle school (%)	10.93	3.60	13.66	3.93	13.14
Expected schooling (year)	18.16	18.26	18.12	18.17	18.16

*Means or percentages were presented in the cells. City variable was not presented in the table*.

Of the respondents, 27.12% of fathers and 20.54% of mothers are only children. In the sample, respondents with siblings do not have a high number of siblings—the fathers with siblings have 0.78 brothers and 1.02 sisters on average, while the mothers with siblings have 0.97 brothers and 0.91 sisters on average. Over 60% of the respondents are sibling children couples, while 25% are only-sibling couples, and 12.7% are only children couples.

Among the covariates measuring parents' resources, it seems that parents of only children tend to have more resources than parents with siblings. Compared to parents with siblings, only-child parents in our sample have higher education and income and are also more likely to come from urban areas and own houses.

The measures of the grandparents' resources in our sample yielded particularly interesting results regarding only daughters. Indeed, the results for the sample illustrate that married only daughters are more likely to receive help from their parents with housework and child care (42.33%) than non-only daughters (15.09%) and are far more likely to live with their parents (30.1%) than non-only daughters (8.37%). The Chi-square tests demonstrated that these differences were significant [Pearson chi-square (1) = 253.69, *p* = 0.000; and Pearson chi-square (1) = 231.33, *p* = 0.000]. Similar results were found in the sample with respect to the proportion of married only sons who accept help from or live with their parents.

Table 2 presents the results of the average educational investment in children by parents' sibship size. According to the results, there seems to be a negative correlation between average educational investment in children and the parents' number of siblings for our sample, with this negative effect slightly higher with respect to mothers' sibship size, compared to that of the fathers. Further distinguishing between the father's and mother's number of siblings reveals a possible negative correlation between educational investment in children and parents' number of brothers, and the mother's number of brothers has a greater negative effect than the father's number of brothers. However, the effect of the father/mother's number of sisters on the educational investment in their children is ambiguous, with only vague evidence of a negative association observable in Table 2. As such, the preliminary results presented in Table 2 reveal the complexity of parents' sibling effects, especially when taking gender into account.

**Table 2:** Average educational investment (Thousand Yuan), by parents' sibship size.

		**Fathers' sibship size**						
		**0**	**1**	**2**	**3**	**4**	**5**	**6+**
Mothers' sibship size	0	26.19	24.37	21.99	23.51	17.96	16.16	30.80
	1	20.31	19.09	19.27	17.40	15.48	14.65	28.85
	2	18.91	17.37	15.68	14.02	12.21	12.65	16.45
	3	22.87	14.98	13.04	13.09	9.69	18.59	12.20
	4	20.82	13.19	14.68	11.54	14.64	21.05	9.83
	5	18.64	15.62	12.30	10.91	10.38	3.00	8.80
	6+	24.90	16.20	12.11	11.16	11.60	7.1	18.48
		**Fathers' number of brothers**						
		**0**	**1**	**2**	**3**	**4**	**5**	**6+**
Mothers' number of brothers	0	23.95	19.97	20.89	15.36	23.08	–	–
	1	18.06	16.89	15.32	13.54	13.69	–	43.60
	2	16.21	14.77	12.25	16.48	13.30	–	–
	3	15.51	14.99	15.48	15.08	10.13	–	–
	4	11.98	5.64	10.90	–	8.80	–	–
	5	–	–	–	–	–	–	–
	6+	9.80	–	–	–	–	–	–
		**Fathers' number of sisters**						
		**0**	**1**	**2**	**3**	**4**	**5**	**6+**
Mothers' number of sisters	0	22.73	19.77	17.45	19.71	16.60	20.05	–
	1	18.47	16.75	16.08	17.83	15.22	15.10	15.60
	2	18.42	15.90	15.43	10.18	17.63	9.83	–
	3	16.13	13.47	10.16	24.83	7.20	–	–
	4	15.23	14.43	12.46	8.09	11.47	–	13.60
	5	24.38	10.59	18.26	–	–	–	–
	6+	8.95	16.9	16.90	–	28.00	–	–

*Most of the parents had 4 or less siblings/brothers/sisters. Thus, the 5 and 6+ raw/column had a small sample size, many cells even 0*.

### The Effects of Parents' Sibship Size

As neglected confounding variables may mask the comparisons above, this study tested the sibship size effects with control variables. Random intercept models were used to control the effect of families nested in cities—families may not be assigned at random to cities. Adopting the family resource dilution approach, this study estimated the effects of the parents' number of siblings on the educational investment in their children. Then, the sibship size was categorized into father's siblings and mother's siblings to explore the impact of gender. Accordingly, Model 1 estimates the effect of parents' sibship size on the educational investment in children, while Model 2 separately examines sibship size with respect to the father's and the mother's number of siblings, respectively. Table 3 presents the results of the two models.

**Table 3:** Estimated effects of sibship size on educational investment in children.

	**Model 1**		**Model 2**	
	**Coefficient**	**s.e**.	**Coefficient**	**s.e**.
Constant	−6.95	4.82	−6.92	4.83
Parents' siblings	−0.52^***^	0.15		
Fathers' siblings			−0.51^*^	0.23
Mothers' siblings			−0.53^*^	0.22
**Parents' resources**				
Age (year)	0.18	0.12	0.18	0.12
Registered residence (Urban = 0)	−0.72	0.54	−0.72	0.54
Schooling (year)	0.54^***^	0.12	0.54^***^	0.12
Occupation (Non-Monopoly = 0)	0.13	0.56	0.13	0.56
Income (Thousand Yuan, CNY)	0.73^***^	0.06	0.73^***^	0.06
Housing (Others = 0)	−0.09	0.54	−0.09	0.54
**Grandparents' resources**				
Fathers' parents' schooling (year)	0.16	0.09	0.16	0.09
Mothers' parents' schooling (year)	0.15	0.10	0.15	0.10
Fathers' parents' help (No = 0)	−1.67^**^	0.55	−1.66^**^	0.55
Mothers' parents' help (No = 0)	−0.26	0.67	−0.26	0.68
Fathers' parents' co-residence (No = 0)	−0.82	0.54	−0.82	0.55
Mothers' parents' co-residence (No = 0)	0.42	0.76	0.41	0.76
**Children's information**				
Gender (Male = 0)	1.03^*^	0.47	1.02^*^	0.47
Age (year)	0.20	0.17	0.20	0.17
Sibling (0 = 0)
1	−0.54	0.60	−0.54	0.60
2	−3.21	1.80	−3.21	1.80
School (Kindergarten = 0)
Primary school	−0.13	0.88	−0.13	0.88
Junior middle School	−1.09	1.67	−1.09	1.67
Expected Schooling (year)	0.24^*^	0.10	0.24^*^	0.10
Var (cons)	22.86	9.72	22.86	9.72
Var (Residual)	173.90	4.37	173.90	4.37
Log-likelihood	−12,715.60		−12,715.60	
*N*	3,175		3,175	
Number of cities	12		12	

* p < 0.05,

** p < 0.01, and

*** *p < 0.001*.

As expected, the results of Model 1 demonstrate that the parents' number of siblings has a notable negative effect on the educational investment in their children, which is in line with this study's first hypothesis. When all other variables are controlled, each increase in the parents' number of siblings reduces the family's educational investment in their children by an average of approximately CNY 500.

Regarding other explanatory variables, the parents' education level and monthly income are significant among parents' resource covariates. Keeping all other variables controlled, when the parents' education increases by 1 year, the family's educational investment in children will increase by approximately CNY 500. Similarly, when the parents' monthly income increases by CNY 1,000, the family's educational investment increases by CNY 730. Meanwhile, the parents' age, registered residence, occupation, and housing exhibited no significant effect on their children's educational investment.

In terms of the grandparents' resource covariates, help provided by the father's parents proved to be a significant variable. Under the same conditions, paternal grandparents' help with housework and child care resulted in the family spending CNY 1,690 less on the educational investment in their children than without such help. This result is largely explained by the fact that help from grandparents constitutes a form of intergenerational resource exchange and typically occurs patrilineally. Meanwhile, grandparents' education, help provided by the mother's parents, and grandparents' coresidence were found to have no significant effect.

Regarding child-related factors, only the variables of the child's gender and the expected years of schooling were found to be significant. Under the same conditions, the average educational expenditure on a son was approximately CNY 1,000 less than that on a daughter. This is largely because the unit price of tutoring classes for girls is consistently higher than for boys. Expected schooling has a positive impact in that for every 1-year increase in expected years of schooling, the child's educational investment increases by CNY 240.

Model 2 estimates the effects of the father's number of siblings and the mother's number of siblings on educational investment in children. Both parents' number of siblings was found to have a negative impact. When all the other variables are controlled, educational expenditure on a child decreases by CNY 510 for each additional sibling of the father and by CNY 530 for each additional sibling of the mother. As such, the results of Model 2 are consistent with those of Model 1 and in line with the study's first hypothesis.

To further analyze the effects of parents' sibship size by gender, this study constructed a third model, Model 3, by dividing the parents' siblings into two groups: father/mother's number of brothers and father/mother's number of sisters. A fourth model, Model 4, divides the parents' siblings by seniority and gender—namely, elder brother, younger brother, elder sister, and younger sister—to further compare sibling effects. Table 4 presents the results of the two models.

**Table 4:** Estimated effects of sibship size, by gender, on educational investment in children.

	**Model 3**		**Model 4**	
	**Coefficient**	**s.e**.	**Coefficient**	**s.e**.
Constant	−6.86	4.84	−6.87	4.86
Fathers' brothers	−0.66	0.35		
Fathers' sisters	−0.35	0.27		
Mothers' brothers	−0.91^*^	0.36		
Mothers' sisters	−0.29	0.26		
Fathers' elder brothers			−0.75	0.39
Fathers' young brothers			−0.67	0.56
Fathers' elder sisters			−0.21	0.29
Fathers' young sisters			−0.93	0.52
Mothers' elder brothers			−0.80	0.42
Mothers' young brothers			−0.95^*^	0.47
Mothers' elder sisters			−0.44	0.31
Mothers' young sisters			0.02	0.41
**Parents' resources**				
Age (year)	0.18	0.12	0.19	0.12
Registered residence (Urban = 0)	−0.71	0.54	−0.68	0.54
Schooling (year)	0.53^***^	0.12	0.53^***^	0.12
Occupation (Non-Monopoly = 0)	0.09	0.56	0.10	0.56
Income (Thousand Yuan, CNY)	0.73^***^	0.06	0.73^***^	0.06
Housing (Others = 0)	−0.08	0.54	−0.11	0.54
**Grandparents' resources**				
Fathers' parents' schooling (year)	0.16	0.09	0.16	0.09
Mothers' parents' schooling (year)	0.15	0.10	0.15	0.10
Fathers' parents' help (No = 0)	−1.69^**^	0.55	−1.70^**^	0.55
Mothers' parents' help (No = 0)	−0.28	0.68	−0.32	0.68
Fathers' parents' co-residence (No = 0)	−0.83	0.55	−0.85	0.55
Mothers' parents' co-residence (No = 0)	0.32	0.76	0.35	0.77
**Children's information**				
Gender (Male = 0)	1.02^*^	0.47	1.01^*^	0.47
Age (year)	0.19	0.17	0.18	0.17
Sibling (0 = 0)				
1	−0.56	0.60	−0.58	0.60
2	−3.17	1.80	−3.13	1.80
School (Kindergarten = 0)				
Primary school	−0.12	0.88	−0.07	0.88
Junior middle school	−1.04	1.67	−0.95	1.67
Expected schooling (year)	0.24^*^	0.10	0.23^*^	0.10
Var (cons)	22.70	9.66	22.47	9.56
Var (Residual)	173.79	4.37	173.65	4.37
Log-likelihood	−12,714.60		−12,713.25	
*N*	3,175		3,175	
Number of cities	12		12	

* p < 0.05,

** p <0.01, and

*** *p < 0.001*.

As Table 4 illustrates, the results of Model 3 reveal that the father's number of siblings does not significantly affect his own children's educational investment. In contrast, the mothers' number of brothers has a negative effect on educational expenditure on her children, while her number of sisters is not significant. When all other variables are controlled, the child's educational investment will decrease by CNY 910 for each additional brother the mother has. The results are in line with this study's second hypothesis. As such, the negative effects of parents' sibship size differ for mothers and fathers, as well as between brothers and sisters.

As noted, Model 4 (Table 4) further divides the father/mother's siblings into father/mother's number of elder brothers, younger brothers, elder sisters, and younger sisters. The results of Model 4 are straightforward: only the mother's number of younger brothers is demonstrated to have a significant negative impact. When all other variables are controlled, the child's educational investment will decrease by CNY 950 for each additional younger brother the mother has. The results confirm that married daughters are more likely to be influenced by siblings and that the detrimental effects are primarily due to their having younger brothers, confirming the third hypothesis of this study.

### The Effects of Parents' Only-Children Status

Models 1–4 also explore the impact of the father/mother's only-child status through the linearity constraint. Naturally, only children experience no negative effects of sibship size. The results of Model 4 (Table 4) indicate that this advantage is particularly important for mothers. To confirm the results of the only-child effect, this study constructed Model 5 by introducing whether the father/mother is an only child as a variable. Table 5 presents the results of this model.

**Table 5:** Estimated effects of parents' only children on educational investment in children.

	**Model 5**	
	**Coefficient**	**s.e**.
Constant	−6.29	4.82
Father only child (only = 0)	−1.21	0.62
Mother only child (only = 0)	−1.41^*^	0.66
**Parents' resources**		
Age (year)	0.17	0.12
Registered residence (Urban = 0)	−0.77	0.54
Schooling (year)	0.55^***^	0.12
Occupation (Non-Monopoly = 0)	0.10	0.56
Income (Thousand Yuan, CNY)	0.74^***^	0.06
Housing (Others = 0)	−0.05	0.54
**Grandparents' resources**		
Fathers' parents' schooling (year)	0.18	0.09
Mothers' parents' schooling (year)	0.15	0.10
Fathers' parents' help (No = 0)	−1.64^**^	0.55
Mothers' parents' help (No = 0)	−0.30	0.68
Fathers' parents' co-residence (No = 0)	−0.79	0.55
Mothers' parents' co-residence (No = 0)	0.36	0.77
**Children's information**		
Gender (Male = 0)	1.06^*^	0.47
Age (year)	0.18	0.17
Sibling (0 = 0)
1	−0.76	0.60
2	−3.75^*^	1.80
School (Kindergarten = 0)		
Primary school	0.07	0.88
Junior middle school	−0.88	1.67
Expected schooling	0.24^*^	0.10
Var (cons)	22.20	9.47
Var (Residual)	174.08	4.38
Log-likelihood	−12,717.03	
*N*	3,175	
Number of cities	12	

* p < 0.05,

** p < 0.01, and

*** *p < 0.001*.

The results of Model 5 are consistent with those of the previous models: while being an only-child father does not have any significant effects, only-child mothers have an advantage. When all other variables are controlled, the educational investment in the child of an only-child mother is about CNY 1,410 higher than that of a mother with siblings, which is in line with this study's fourth hypothesis. Therefore, in the Chinese context—where sons typically receive preferential treatment—the number of siblings will be less important for sons. In this respect, families will often guarantee the attainment of sons at the cost of opportunities for daughters. The results of this study indicate that this is more likely to occur when the daughters have many younger brothers. This effect is especially evident when the daughters get married and, traditionally speaking, are no longer considered a member of the family. In contrast, as their parents' only child, married only daughters retain their connection to their parents' resources.

## Discussion

This study explored family resource dilution in Chinese expanded families with respect to the country's generation of only children and whether only daughters remain empowered as they marry and have children of their own. Based on data from the “Study of Youths in 12 Cities of Mainland China,” this study examined educational investment in children—an important component of family finance in which two generations (i.e., the fathers/mothers and the parents of the fathers/mothers) are typically involved, with four hypotheses. In doing so, this study found empirical evidence that the dilution of family resources in Chinese expanded families still benefits men and remains patrilineal, which confirmed our first and second hypotheses. Son preference remains prevalent in Chinese families, leading to many parents sacrificing the interests of their daughters to ensure support for their sons. Accordingly, the dilution effect of family resources has a more significant negative effect on daughters than on sons—even after the sons and daughters marry and have children of their own. The results of this study demonstrate that mothers are more likely to be affected by the number of siblings, especially with respect to younger brothers, confirming our third hypothesis. In contrast, married only daughters retain a position of empowerment. When only daughters marry and leave home, they retain their connection to their parents' resources, giving them a relatively dominant position regarding the educational expenditure on their children. This study's results indicate that only-child mothers have a significant advantage over mothers with siblings, which confirmed the fourth hypothesis.

The results of this study contribute to the field of Chinese only-children studies; this study also comments on the approach of preceding research. As the phenomenon of only children in China is mainly a result of the OCP, especially at the beginning, research has focused on the differences between only children and children with siblings, particularly with respect to the socialization process of Chinese only children. However, this study suggests that the research should also focus on the only children who are now grown up, have left their parents' homes, and have formed their own families. As this study demonstrates, family structure has a clear impact on only children, making it necessary to study only children from the perspective of the family, especially the “expanded family,” in China.

This study further suggests that the family resource dilution theory should be extended to China's expanded families. As a concise paradigm, the family resource dilution theory effectively explains the negative relationship between children's development and the number of children in a family. However, the family in this theory is modeled on the nuclear family. Chinese families differ from those of America and Europe, and researchers need to consider such specificity. Scholars have attempted to incorporate the gender structure of Chinese families into family resource dilution theory. For instance, a study in Taiwan, Republic of China, indicates that the dilution of resources in Chinese families prioritizes sons, with daughters—especially older sisters—typically being placed in a disadvantageous position (Chu et al., 2007). Extant research indicates that only children also need to be considered, particularly insofar as only daughters reveal the traditional neglect of girls in Chinese families (Fong, 2002, 2004; Wang, 2011a,b). This study demonstrates that the family resource dilution theory can be applied to three-generation families. As intergenerational relationships in Chinese families tend to be quite close, the expanded family will also experience the effects of resource dilution. Certainly, this study's results indicate that the higher the number of younger brothers a married daughter has, the less educational investment will be available for her children.

The findings of this study have some implications in terms of social policy. Since the OCP was established, the Chinese government has introduced many policies to support only children, and when the Chinese government began to modify the OCP, it started from only-child couples. However, as this study demonstrates, Chinese only children have an advantage in obtaining family resources, especially the only daughters—both in their parents' family and in their own family. However, as the first generation of only children has not yet faced, for example, the heavy burden of caring for their older relatives—which has been a concern of the government—at this stage, relevant policies should pay greater attention to sibling-child families, and especially the gender difference of siblings. As China has entered the new era of the Two-Child Policy, some couples will choose to have two children in their families, which will undoubtedly increase social policies that accommodate sibling children.

This study has some limitations. First, the measure of its dependent variable, educational investment in children, is fairly broad, particularly as this study does not distinguish between different types of spending in basic education and other forms of education—such as tutoring classes—and only considers the total educational expenditure. Future studies should deconstruct educational expenditure, as this will likely shed further light on the topic. Second, while this study discusses grandparents' educational investment in their grandchildren, it does not include direct measures of this investment, simply assuming that the grandparents are heavily involved in the educational investment of the family, according to Chinese convention. This may result in an overestimation of the study hypothesis. Accordingly, future research should focus directly on the grandparents to clarify their investment in their children's family and their grandchildren. In this respect, qualitative research may prove beneficial in expanding on the conclusions drawn in this study.

In summary, with a large and representative sample of Chinese adult only children and children with siblings, using educational investment in children as an example, this study provided reliable evidence that Chinese families tend to sacrifice the interests of daughters to ensure support for sons, even when the children are married and have children of their own. Additionally, the present study confirmed that married only daughters have a relatively dominant position in decisions regarding the family's educational expenditure on her children. These findings confirm that both the theory of family resources dilution and gender difference are of great significance in Chinese expanded families.

## Data Availability Statement

The raw data supporting the conclusions of this article will be made available by the authors, without undue reservation.

## Ethics Statement

Ethical review and approval was not required for the study on human participants in accordance with the local legislation and institutional requirements. The patients/participants provided their written informed consent to participate in this study.

## Author Contributions

All authors listed have made a substantial, direct and intellectual contribution to the work, and approved it for publication.

## Conflict of Interest

The authors declare that the research was conducted in the absence of any commercial or financial relationships that could be construed as a potential conflict of interest.
